# Exploring the relationship between coronary heart disease and type 2 diabetes: a cross-sectional study of secondary prevention among diabetes patients

**DOI:** 10.3399/bjgpopen18X101636

**Published:** 2019-03-20

**Authors:** Bjørn Gjelsvik, Anh Thi Tran, Tore J Berg, Åsne Bakke, Ibrahimu Mdala, Kjersti Nøkleby, John G Cooper, Tor Claudi, Karianne Fjeld Løvaas, Geir Thue, Sverre Sandberg, Anne K Jenum

**Affiliations:** 1 Assistant Professor, Department of General Practice, General Practice Research Unit (AFE), Institute of Health and Society, University of Oslo, Oslo, Norway; 2 Researcher, Department of General Practice, General Practice Research Unit (AFE), Institute of Health and Society, University of Oslo, Oslo, Norway; 3 Assistant Professor, Institute of Clinical Medicine, Faculty of Medicine, University of Oslo, Oslo, Norway; 4 Senior Consultant, Department of Endocrinology, Morbid Obesity and Preventive Medicine, Oslo University Hospital, Oslo, Norway; 5 Senior Consultant, Department of Medicine, Stavanger University Hospital, Stavanger, Norway; 6 PhD Fellow, Department of Global Public Health and Primary Care, University of Bergen, Bergen, Norway; 7 Statistician, Department of General Practice, General Practice Research Unit (AFE), Institute of Health and Society, University of Oslo, Oslo, Norway; 8 PhD Fellow, Department of General Practice, General Practice Research Unit (AFE), Institute of Health and Society, University of Oslo, Oslo, Norway; 9 Researcher, Norwegian Quality Improvement of Laboratory Examinations, Haraldsplass Deaconess Hospital, Bergen, Norway; 10 Senior Consultant, Department of Medicine, Stavanger University Hospital, Stavanger, Norway; 11 Senior Consultant, Department of Medicine, Nordland Hospital, Bodø, Norway; 12 Section Head, Norwegian Quality Improvement of Laboratory Examinations, Haraldsplass Deaconess Hospital, Bergen, Norway; 13 Professor, Department of Global Public Health and Primary Care, University of Bergen, Bergen, Norway; 14 Professor, Norwegian Quality Improvement of Laboratory Examinations, Haraldsplass Deaconess Hospital, Bergen, Norway; 15 Professor, Laboratory of Clinical Biochemistry, Haukeland University Hospital, Bergen, Norway; 16 Professor, Department of Global Public Health and Primary Care, University of Bergen, Bergen, Norway; 17 Director, Norwegian Quality Improvement of Laboratory Examinations, Haraldsplass Deaconess Hospital, Bergen, Norway; 18 Professor, Department of General Practice, General Practice Research Unit (AFE), Institute of Health and Society, University of Oslo, Oslo, Norway

**Keywords:** type 2 diabetes, coronary heart disease, stroke, secondary prevention, general practice, primary care

## Abstract

**Background:**

Coronary heart disease (CHD) and stroke are the major causes of death among people with diabetes.

**Aim:**

To describe the prevalence and onset of CHD and stroke among patients with type 2 diabetes mellitus (T2DM) in primary care in Norway, and explore the quality of secondary prevention.

**Design & setting:**

A cross-sectional study of data was undertaken from electronic medical records (EMRs) of 10 255 patients with T2DM in general practice. The study took place in five counties of Norway (Oslo, Akershus, Rogaland, Hordaland, and Nordland). Quality of care was assessed based on national guideline recommendations.

**Method:**

Summary statistics with adjustments and binary logistic regression models were used.

**Results:**

In total, 2260 patients (22.1%) had CHD and 759 (7.4%) had stroke. South Asians had significantly more CHD than ethnic Norwegians (29.5%, 95% confidence interval [CI] = 26.1 to 33.0 versus 21.5%, CI = 20.6 to 22.3) and other ethnic groups, and experienced onset of CHD or stroke at a mean of 7 years before Norwegians. In 47.9% of the patients, CHD was diagnosed before T2DM. Treatment target for low-density lipoprotein (LDL) cholesterol was reached for 30.0% and for systolic blood pressure (SBP) for 65.1% of the patients with CHD. Further, 20.9% of patients with CHD were present smokers, and only 5.0% of patients reached all four treatment targets (no smoking, HbA1c ≤7.0%, SBP <135 mmHg, LDL-cholesterol <1.8 mmol/l).

**Conclusion:**

The diagnosis of CHD preceded the diagnosis of T2DM in half of the patients. The prevalence of CHD was highest and onset earlier among ethnic South Asians. More intensive treatment of lipids, blood pressure, and smoking are needed in patients with T2DM and CHD.

## How this fits in

CHD and stroke are prevalent among people with T2DM, and are generally thought to be a complication of diabetes. However, this survey found that diagnosis of CHD preceded the diagnosis of T2DM in half of the patients. An increased prevalence and earlier onset of CHD was also found among people of South Asian ethnicity. Only 30.0% of CHD patients reached treatment target for LDL-cholesterol, and more intensive care is needed for people with multiple elevated risk factors. 

## Introduction

CHD and stroke are the major causes of death among people with diabetes.^1^ T2DM has been associated with a doubling of the risk for CHD and stroke,^2^ although a somewhat lower risk has recently been reported in Scandinavia.^3,4^ Cardiovascular disease (CVD) among patients with T2DM places pressure on the healthcare system,^5,6^ but multifactorial secondary prevention reduces morbidity and years of life lost.^7–9^

The influence of chronic hyperglycaemia on atherosclerosis is not fully understood,^10^ and studies describing the relation between the onset of CVD and of T2DM are few. There has, however, been an increasing awareness of diagnosing T2DM among patients with CHD. Recently, statins have been shown to exert a diabetogenic effect,^11,12^ which also may contribute to an increasing prevalence of T2DM among patients with CHD.

GPs follow up most patients with T2DM in Norway; therefore, the quality of care in general practice is essential for the clinical outcomes of these patients.

The aims in the present article were: firstly, to describe the prevalence of CHD and stroke among patients with T2DM, time of onset, and distribution according to age, sex, ethnic group, and region in a primary care setting in Norway; and secondly, to characterise the secondary preventive efforts among T2DM patients with CHD and stroke to identify potential treatment gaps, and identify patient and GP factors associated with successful achievement of treatment goals.

## Method

The study is part of the ROSA 4 study, which is a cross-sectional survey assessing the quality of care for patients with diabetes in general practice in Norway. In total, 106 practices with 367 GPs in five counties of Norway (Oslo, Akershus, Rogaland, Hordaland, and Nordland) were invited to the study. Detailed information about the method is available elsewhere.^13^

In short, a software program (Noklus) was used to identify all patients aged ≥18 years with a diabetes diagnosis (using the ICPC-2 codes: T89 for diabetes type 1 and T90 for diabetes type 2) recorded from 2012–2014, and to capture pre-defined data from EMRs. Research nurses examined the EMRs to verify the electronically registered data and to collect other relevant data regarding diabetes care. A questionnaire was used to gather GPs' self-reported characteristics such as age, sex, and specialist status.

Variables used in the present study include the following: patient characteristics (such as age, sex, year of diabetes diagnosis, height, and weight); smoking status; pharmacological therapy; intermediate outcomes (including HbA1c, blood pressure, total cholesterol, LDL-cholesterol, high-density lipoprotein [HDL]-cholesterol and triglycerides); macrovascular complications (prevalence and year for diagnosis): CHD (angina, myocardial infarction, percutaneous coronary intervention/coronary artery bypass surgery), stroke (excluding transient ischaemic attacks); and atrial fibrillation (AF). For the majority of variables, the most recently recorded value from 1 October 2013–31 December 2014 was used; for smoking habits, the period was 1 January 2010–31 December 2014.

Further, Statistics Norway supplied information about country of birth and educational level. The patient’s ethnic group was based on country of birth and was categorised as: (1) Norwegian (born in Norway); (2) South Asian (born in Pakistan, Sri Lanka, India, and Bangladesh); and (3) other. The patient’s education was grouped into: (1) primary or no education; (2) high school and/or vocational training; and (3) university education.

The quality of care was assessed according to key recommendations for treatment and treatment targets in the Norwegian guideline at the time of the survey; for example, the treatment target for HbA1c was ≤7.0% (53 mmol/mol), intervention threshold for blood pressure was >140/85 mmHg with treatment targets ≤135/80 mmHg. In patients with CHD, the treatment target for LDL was <1.8 mmol/l. Cardioprotective treatment with acetylsalicylic acid (ASA, commonly known as aspirin) 75 mg was recommended for T2DM with CHD.^14^

### Statistical analyses

Crude prevalence of macrovascular complications and average adjusted prevalence with 95% CI stratified by sex, ethnic group, and county are reported.

Pharmacological treatment and intermediate outcomes (HbA1c, BP, and lipids) are reported for patients with CHD and stroke respectively. Descriptive statistics in the form of proportions, means (with standard deviations), or median values (with percentiles) were used to describe the patient characteristics by the stratification variables. Independent sample *t*-tests and analysis of variance (ANOVA) were used to compare mean differences of numerical variables between different patient groups. Associations between categorical factors were established from the χ^2^ tests.

The generalised estimating equation (GEE) binary logistic regression models with random effects at practice level were used to identify factors that were associated with achievement of treatment goals. Similar models were fitted to binary data on prevalence of CHD before and after the diagnosis of T2DM. All models were adjusted for patient-level characteristics (for example, age, sex, ethnic group, and education) while further adjustments using GP-level characteristics (for example, sex, and specialist status) were done in models assessing treatment goals. The analyses were performed with SPSS (version 24) and StataSE (version 15). Owing to multiple testing, the significance level was adjusted accordingly, based on the Bonferroni correction.

## Results

Seventy-seven practices (72.7% of those invited) with 282 GPs (76.3% of those invited) agreed to participate, and provided data for 10 255 patients with T2DM. The baseline characteristics and intermediate outcomes of these patients stratified according to sex and presence or absence of CHD and stroke are presented in Table 1.

### Prevalence of CHD, stroke, and AF

**Figure 1.**
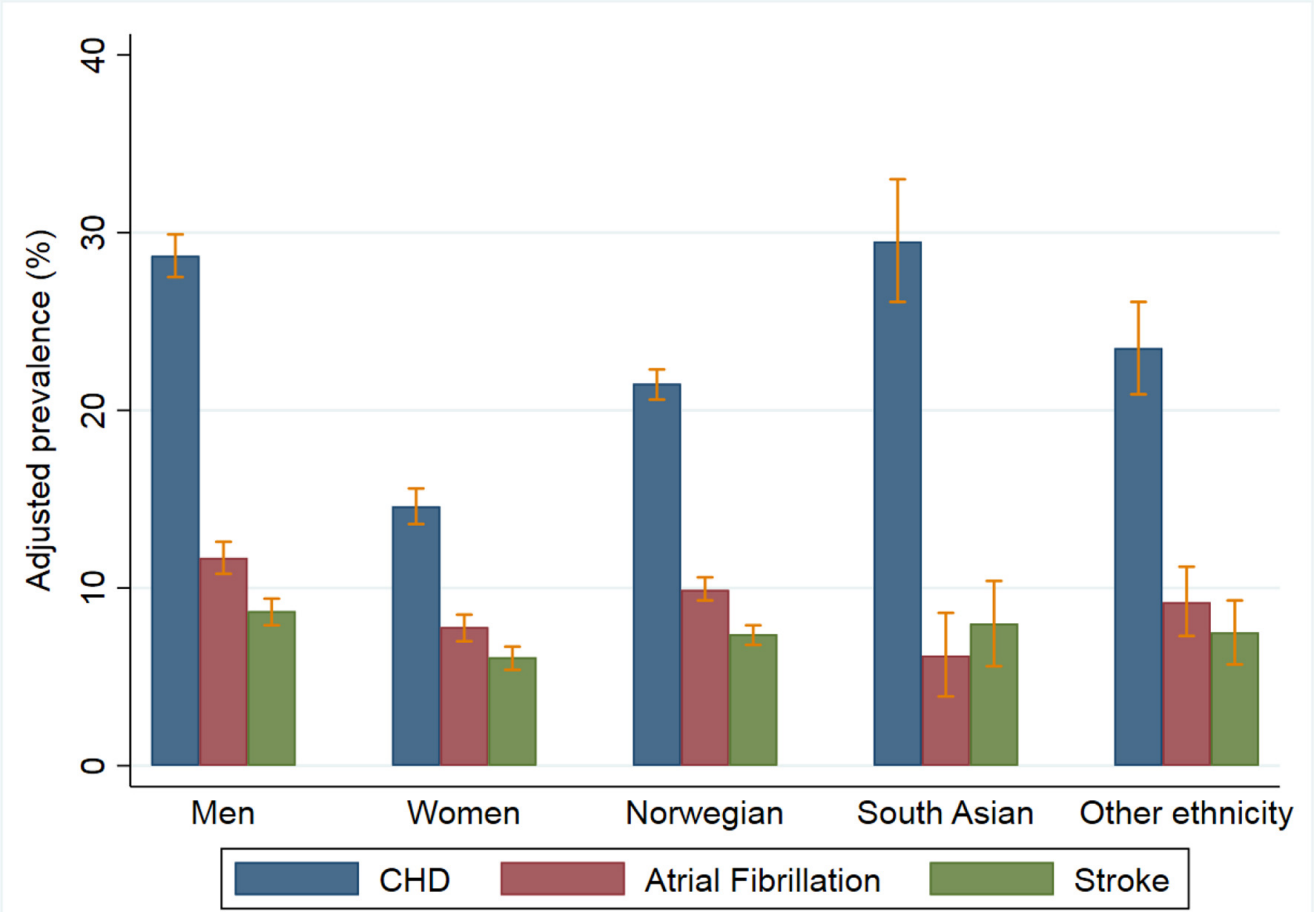
Adjusted prevalence of CHD, atrial fibrillation, and stroke by sex and ethnic group. Numbers for sex are adjusted for age and clustering within practices. Numbers for ethnic group are adjusted for age, sex, and clustering (see Table 1). CHD = coronary heart disease.

In total, 2260 patients (22.1%, missing data = 25) had CHD and 759 (7.4%, missing data = 9) had stroke reported in their EMRs (Table 1). Taken together, 30.9% of the patients had CHD, stroke, or AF, or any combination of the three. The adjusted prevalence of CHD for males was twice the prevalence among females (28.7% versus 14.6%), and there were significant differences between ethnic groups (Figure 1). These differences started at a young age and increased up to 64 years (further information available from the authors on request). The same sex difference, although to a lesser extent, was observed for stroke and AF. The mean age of onset for CHD and stroke was 7 years earlier in South Asians than in ethnic Norwegians. The prevalence of stroke was lower in Rogaland compared with the other counties.

**Figure 2.**
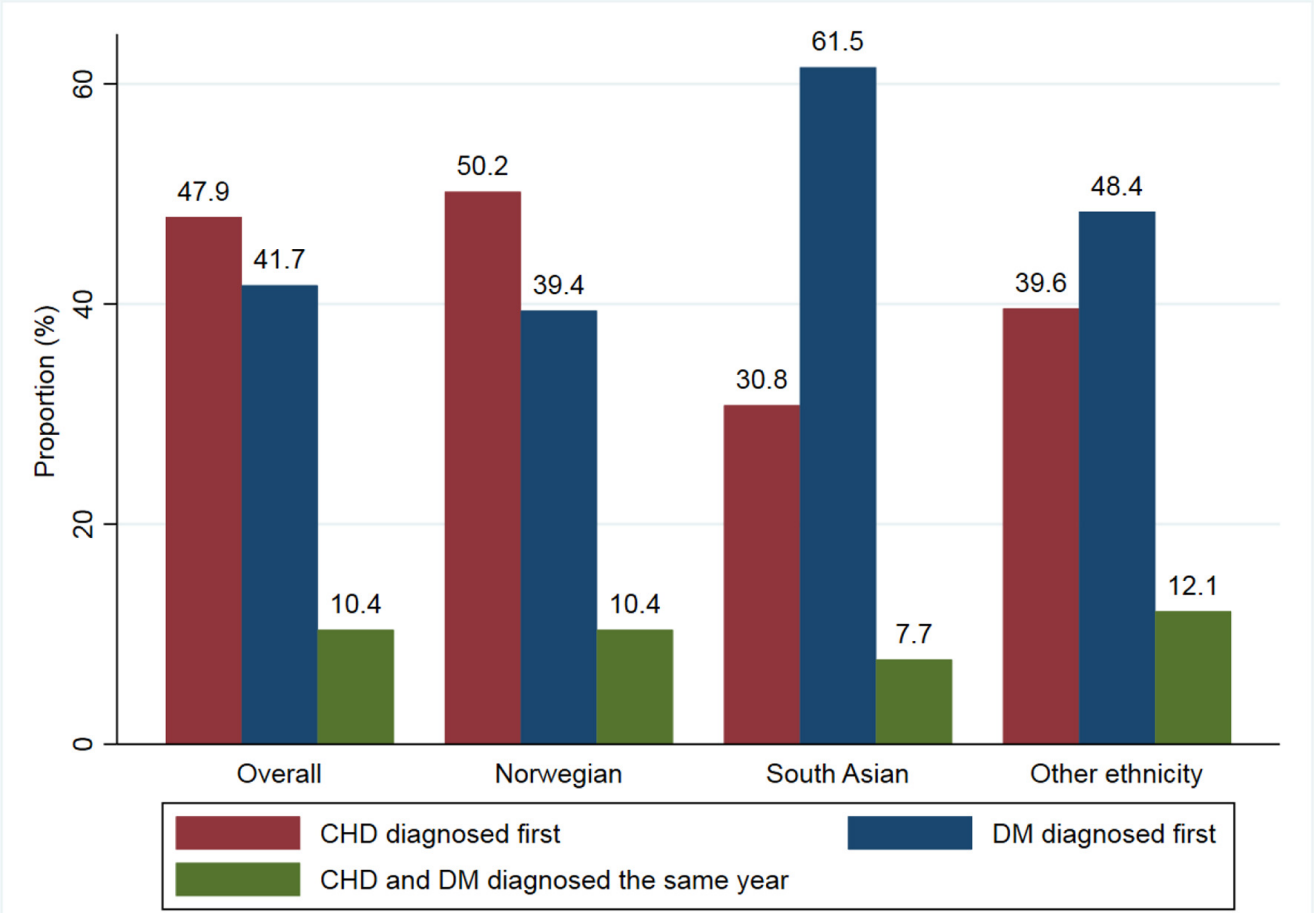
Proportions of patients where CHD was diagnosed ≥1 year previously, the same year, and ≥1 year after the diagnosis of type 2 diabetes (overall and in different ethnic groups) CHD = coronary heart disease. DM = diabetes mellitus.

**Table 1.** Prevalence of coronary heart disease (angina, cardiac infarction, or PCI/bypass), stroke, and atrial fibrillation in patients with type 2 diabetes, stratified by sex, ethnic group, and county (results from the ROSA 4 study, 2014)

	Total^a^	Sex^b^		Ethnicity^c,d^			County^e^				
		Females	Males	Norwegian	South Asian	Other	Nordland	Hordaland	Rogaland	Akershus	Oslo
**CHD**											
*n* (prevalence,%)	2260 (22.1)	727 (15.8)	1533 (27.3)	1891 (23.0)	161 (20.2)	108 (17.2)	675 (24.2)	390 (24.3)	386 (20.4)	279 (19.7)	530 (21)
Adjusted prevalence (95% CI)^a^	22.1(21.2 to 22.9)	14.6^f ^ (13.6 to 15.6)	28.7(27.5 to 29.9)	21.5(20.6 to 22.3)	29.5^g ^ (26.1 to 33.0)	23.5(20.9 to 26.1)	23.8(22.4 to 25.3)	23.9 (22.0 to 25.8)	21.2(19.4 to 22.9)	18.9(17.0 to 20.8)	21.5(20.0 to 23.0)
Mean age for CHD diagnosis (SD)	60.1 (11.3)	64.3^f ^(11.5)	58.2 (10.6)	61 (11.2)	53.1^g ^(9.7)	58.2^g ^(11.1)	60.4 (11)	59.8 (11.1)	59.6 (11.2)	62.5 (10.8)	59.1 (11.8)
**Stroke**											
*n* (prevalence,%)	759 (7.4)	308 (6.7)	451 (8.0)	658 (8.0)	38 (4.8)	62 (5.1)	213 (7.6)	146 (9.1)	101 (5.3)	99 (7.0)	200 (7.9)
Adjusted prevalence (95% CI)^a^	7.4 (6.9 to 7.9)	6.1^f ^ (5.4 to 6.7)	8.7 (7.9 to 9.4)	7.4 (6.8 to 7.9)	8.0 (5.6 to 10.4)	7.5 (5.7 to 9.3)	7.3 (6.4 to 8.2)	8.8 (7.5 to 10.1)	5.6^h ^ (4.6 to 6.6)	6.6 (5.4 to 7.8)	8.5 (7.4 to 9.6)
Mean age for stroke diagnosis (SD)	64.5 (12.8)	67.7**^f ^**(12.6)	62.3 (12.4)	65.3 (12.5)	58.3**^g ^**(11.6)	59.9**^g ^**(13.7)	64.5 (12.6)	64.6 (13.3)	63.5 (12.4)	66.8 (12.3)	63.8 (12.9)
**Atrial fibrillation**											
*n* (prevalence,%)	995 (9.7)	404 (8.8)	591 (10.5)	901 (11.0)	24 (3.0)	69 (5.7)	289 (10.4)	162 (10.1)	157 (8.3)	152 (10.7)	235 (9.3)
Adjusted prevalence (95% CI)^a^	9.7(9.2 to 10.3)	7.8^f ^ (7.0 to 8 5)	11.7(10.8 to 12.6)	9.9(9.3 to 10.6)	6.2^g ^ (3.9 to 8.6)	9.2(7.3 to 11.2)	9.7(8.6 to 10.7)	9.5(8.1 to 10.9)	8.8 (7.5 to 10.0)	10.0(8.5 to 11.6)	10.6(9.3 to 11.9)

The GEE logistic regression was used to adjust for differences in prevalence between groups. The significance level was set at 0.017 based on the Bonferroni correction. Mean differences between groups were analysed with independent sample *t*-test and ANOVA.

^a^Total prevalence (valid per cent) adjusted for clustering between practices. ^b^Sex adjusted for clustering and age; ^c^Ethnic group adjusted for clustering, age, and sex. ^d^Ethnic groups: (1) Norwegian (born in Norway); (2) South Asian (born in Pakistan, India, Sri Lanka, and Bangladesh); and (3) other (born in other countries). ^e^County adjusted for clustering, age, sex, and ethnic group. ^f^ *P*<0.017 – difference between sex (reference = males). ^g^ *P<*0.017 – difference between ethnic groups (reference = Norwegian). ^h^ *P*<0.017 – difference between counties (reference = Oslo).

ANOVA = analysis of variance. CHD = coronary heart disease. GEE = generalised estimating equations. PCI = percutaneous coronary intervention (blocking). SD = standard deviation.

### Relation between age of onset of CHD and T2DM

The diagnosis of CHD preceded the diagnosis of T2DM by ≥1 year for 50.2% of the Norwegian patients and 30.8% of the South Asian patients (Figure 2). The patients with CHD before T2DM were older, more often male, more often smokers, and had a lower educational level compared with the patients with CHD diagnosed after their T2DM diagnosis (additional information available from the author on request). A similar trend was observed for stroke, although the mean age of diagnosis for stroke was 1.8 years after the diagnosis of T2DM. For South Asians, CHD and stroke were diagnosed on average 4.3 years and 7.8 years after T2DM diagnosis respectively.

**Table 2.** Proportion of patients with type 2 diabetes attaining treatment targets^a^ for HbA1c, SBP, lipids, and smoking, stratified by patient status regarding CHD and stroke

Attained targets^a^			With CHD, %(*n *= 2260)	Without CHD, %(*n *= 7970)	With stroke, %(*n *= 759)	Without stroke, %(*n *= 9480)
**Targets and other cut-off values**		**Valid cases (%)**				
**HbA1c^a^**						
≤ 7.0% (≤ 53 mmol/mol)		89	58.6^c^	62.7	61.4	61.8
> 9.0% (>75 mmol/mol)			6.7	6.1	6.7	6.1
**Systolic blood pressure (SBP)^a^**						
Percent attaining overall SBP target		74	65.1	65.7	66.7	65.6
SBP >140 mmHg		87	29.0	28.6	27.4	28.8
**Lipids^a^**						
LDL-cholesterol ≤1.8(mmol/l)		68	30	-	-	-
LDL-cholesterol <2.5(mmol/l)		68	67.9^c^	41.5	64.3^c^	46.2
**Lifestyle**						
No daily smoking		83	79.1^b^	76.9	76.4	77.5
**Proportions achieving specified number of targets**						
	Achieving no target		8.0	10.8	8.4	8.9
	Achieving one target		92.0	90.5	91.6	91.1
	Achieving two targets		63.3	64.9	66.7	66.3
	Achieving three targets		27.9	29.8	33.7	31.7
	Achieving four targets		5.0	6.0	7.9	7.0

Significance tests used are χ2 tests for categorical variables. ^a^Treatment targets for patients with CHD are: HbA1c <7.0%, SBP target <135 mmHg medicated or <140 mmHg unmedicated, LDL-cholesterol <1.8 mmol/l, no smoking. For patients without CHD, the intervention threshold for LDL-cholesterol are LDL >3.5 mmol/l, with treatment target LDL <2.5 mmol/l. ^b^ *P*<0.05, ^c^ *P*<0.001.

CHD = coronary heart disease. LDL = low-density lipoprotein. SBP = systolic blood pressure.

### Intermediary outcomes, treatment, and achievement of treatment targets for secondary prevention of CHD and stroke

Only 5% of patients reached all four targets: HbA1c <7.0%, SBP <135 mmHg or unmedicated <140 mmHg, LDL <1.8 mmol/l, and no smoking (Table 2). The treatment target for HbA1c was reached for 58.6% of the patients with CHD, while 65.1% achieved the SBP target. Among those who did not reach the SBP target, 14.3% were not prescribed antihypertensive medication by their GPs (Table 3). Treatment target for LDL-cholesterol (<1.8 mmol/l) was reached for 30.0% of the CHD patients. A total of 77.3% of patients with CHD and 67.3% of patients with stroke used lipid-lowering agents. In total, 20.9% of the CHD patients were daily smokers. When patients aged >80 years were excluded, only minor changes in the percentages for treatment targets were observed (data not shown).

**Table 3.** Prescriptions during the last 15 months for primary and secondary prevention in patients with type 2 diabetes, CHD, and stroke.

Treatment		Primary prevention			Secondary prevention	
		No CVD, % *n* = 7511	Females, % *n* = 3687	Males, % *n* = 3824	CHD, % *n* = 2260	Stroke, % *n* = 759
Attained SBP targets		65.7	64.1	67.0^a^	65.1	66.7
**Blood pressure medication**						
	Thiazides	26.4	28.2	23.9	25.8	26.7
	ACE inhibitors or aII-receptor blockers	47.7	48.7	46.8	61.5^b^	58.0^b^
	Calcium channel blockers (dihydropyridines)	23.1	22.3	23.8	29.1^b^	34.1^b^
	Beta-blockers	16.9	19.0	14.9^b^	65.6^b^	46.1^b^
	Other BP medication	1.6	1.2	2.0^a^	2.9^b^	2.2
	Mean number of BP medications (SD)	1.2 (1.3)	1.3	1.2	2.1 (1.4)^d^	1.9 (1.4)^d^
	No BP medication	42.8	39.5	46.0^b^	15.8^b^	21.6^b^
	Patients with SBP above target, and not prescribed medication	26.9	-	-	14.3	17.3
**Attainment of LDL target**		-	-	-	30	-
**Lipid-lowering medication**						
	Statin	45.5	45.6	45.4	76.7^b^	66.8^b^
	Ezetimibe	1.6	1.9	1.3^a^	4.7^b^	2.6
	No lipid-lowering medication	53.9	53.7	54.0	22.7^b^	32.7^b^
**Anti-platelet therapy**		22.8	21.6	24.1^a^	74.6^b^	66.3^b^

Significance test performed with Poisson regression analysis comparing medication for males versus females, CHD versus non-CHD patients, and stroke versus non-stroke patients, respectively.

^a^ *P*<0.05. ^b^ *P<0.001.* Significance test performed with χ^2^ test comparing males versus females, CHD versus non-CHD, stroke versus non-stroke, respectively.^c^ *P*<0.05.^d^ *P*<0.001.

ACE = angiotensin-converting enzyme. BP = blood pressure. CHD = coronary heart disease. CVD = cardiovascular disease. LDL = low-density lipoprotein. SBP = systolic blood pressure. SD = standard deviation.

Patients with coexisting T2DM and CHD had more intensive antihypertensive treatment than those without CHD (Table 3). Beta-blockers, angiotensin-converting enzyme (ACE) inhibitors, angiotensin receptor blockers, and calcium channel blockers were more widely used in these patients, while the proportion using thiazides was approximately equal in the two groups. Of the patients with CHD, 74.6% used ASA.

In the models adjusted for confounders, females had an odds ratio (OR) of 0.64 (0.5 to 0.82) (reference = males) for reaching treatment targets for LDL-cholesterol. However, females had an OR of 1.42 (1.16 to 1.74) for reaching SBP target (Table 4). Patients in the north of Norway (Nordland) also had lower odds for reaching the LDL-cholesterol target compared with other counties. People of South Asian and other ethnic groups had an OR of 0.42 (0.29 to 0.7) for reaching the HbA1c target compared with ethnic Norwegians. People with a university degree had higher odds for reaching the HbA1c and no smoking targets.

**Table 4.** Associations between patient factors (including county of residence and education) and factors related to the GP, and the probability of achieving treatment targets for intermediate outcomes in patients with CHD and T2DM.^a^

Covariates	SBP ≤135 or <140^b^	LDL-cholesterol ≤1.8 mmol/l	HbA1c ≤7.0%	No daily smoking
	OR (95% CI)	OR (95% CI)	OR (95% CI)	OR (95% CI)
**Patient factors**				
Age (per one year)	1.03 (1.02 to 1.04)^d^	1.00 (0.99 to 1.01)	1.00 (0.99 to 1.01)	1.06 (1.05 to 1.08)^d^
Sex (male = reference)	1.42 (1.16 to 1.74)^d^	0.64 (0.50 to 0.82)^d^	1.11 (0.92 to 1.35)	0.97 (0.73 to 1.28)
**Socioeconomic factors**				
**County (Oslo = reference)**				
Akershus	0.98 (0.59 to 1.61)	0.79 (0.54 to 1.15)	0.98 (0.65 to 1.47)	1.14 (0.75 to 1.72)
Rogaland	1.05 (0.61 to 1.78)	0.94 (0.67 to 1.33)	1.07 (0.81 to 1.44)	1.11 (0.74 to 1.67)
Hordaland	1.02 (0.61 to 1.72)	0.97 (0.71 to 1.34)	0.94 (0.67 to 1.32)	0.89 (0.59 to 1.34)
Nordland	1.41 (0.85 to 2.35)	0.60 (0.42 to 0.85)^d^	0.94 (0.74 to 1.19)	1.26 (0.83 to 1.93)
**Education (Primary/no education = reference)**				
Secondary education	1.08 (0.86 to 1.35)	1.10 (0.86 to 1.40)	1.12 (0.94 to 1.34)	1.26 (0.96 to 1.65)
University	0.95 (0.67 to 1.33)	1.14 (0.83 to 1.56)	1.34 (1.02 to 1.76)^c^	1.76 (1.18 to 2.61)^c^
**Ethnic group (Norwegian = reference)**				
South Asian	0.94 (0.63 to 1.41)	1.21 (0.82 to 1.80)	0.45 (0.29 to 0.7)^d^	1.91 (1.19 to 3.06)^c^
Other	1.05 (.72 to 1.54)	1.16 (0.79 to 1.58)	0.60 (0.42 to 0.86)^c^	1.06 (0.67 to 1.68)
**GP factors**				
**Specialty (Yes = reference)**				
No	1.24 (0.91 to 1.69)	0.95 (0.72 to 1.26)	0.92 (0.76 to 1.13)	1.15 (0.85 to 1.55)
**Sex (Males = reference)**				
Females	0.81 (0.59 to 1.11)	1.20 (0.91 to 1.57)	1.02 (0.85 to 1.22)	1.34 (1.05 to 1.70)^c^

^a^Multilevel binary logistic regression analyses with four dependent variables in 2260 T2DM patients with CHD, adjusted for clustering between practices. ^b^Systolic blood pressure targets: <140 mmHg for patients not using antihypertensives, and <135 mmHg when medication is prescribed. ^c^ *P*<0.05. *^d^P*<0.001.

CI = confidence intervals. CHD = coronary heart disease. LDL = low-density lipoprotein. OR = odds ratio. SBP = systolic blood pressure. T2DM = type 2 diabetes mellitus.

## Discussion

### Summary

In this study, about one-third of the patients with T2DM had coexisting CHD, stroke, or AF, or a combination of these, and thus were candidates for secondary prevention. South Asians had the highest prevalence of CHD, and had the first event 7 years earlier than ethnic Norwegians. An important and novel finding is that in 50.2% of Norwegian patients with CHD the diagnosis of CHD preceded the diagnosis of T2DM by ≥1 year. Treatment target for LDL-cholesterol was reached for 30.0% and for SBP for 65.1% of the patients with CHD. Further, 20.9% of patients with CHD were present smokers, and only 5.0% of patients reached all four treatment targets (no smoking, HbA1c <7.0%, SBP <135 mmHg, LDL-cholesterol <1.8 mmol/l).

For the patients with CHD, it is not obvious that CHD is a complication of T2DM. As the study is cross-sectional, possible explanations can only be speculated about. The trends for obesity and successive T2DM by ageing may be a factor, also among patients with CVD. CHD and T2DM have in common risk factors such as obesity, hypertension, and dyslipidaemia. However, patients where diagnosis of CHD preceded the diagnosis of T2DM were more often male, were older, and more often smokers or former smokers. Prevalent pre-diabetes or undiagnosed diabetes might also lead to CHD before the diagnosis of T2DM. Another possible explanation could be a hyperglycaemic effect of drugs used for secondary prevention of CHD (such as thiazides). Recently, statin treatment has been reported to exert such effects.^11,12^

Prescriptions for secondary prevention in patients with T2DM is largely in line with national recommendations with regard to medications in use. Of note, the most apparent gap in the quality of secondary prevention was that only 30.0% of patients with CHD reached the treatment target for LDL-cholesterol, despite more frequent use of statins than in 2005.^13^ Still, 31.9% of the patients with CHD did not have the LDL-cholesterol measured within the preceding 15 months. Among those who did not achieve the LDL-cholesterol goal, 19.4% did not use lipid-lowering drugs. Side effects of statins, or fear thereof, might have led to lower adherence to statin therapy or to the prescription of insufficient doses.^15^ It is also worth noting that, despite more prescription of antihypertensive drugs compared with the previous ROSA 3 survey in 2005,^13^ a substantial number of patients with CHD and stroke were still undertreated for high blood pressure.

### Strengths and limitations

The ROSA 4 study is the largest study of patients with T2DM in Norway. The selection of practices from three out of four health regions of Norway indicates that the sample is fairly representative for patients with T2DM treated by Norwegian GPs. The invited practices varied in size, and both urban and rural practices were included. The proportion of GPs with a specialist approval was somewhat higher than among all GPs in Norway (67.5% versus 57.1% ). The mean number of patients on GPs' list was close to the mean for all GPs in Norway. Trained nurses manually validated the diagnosis of T2DM and CVD captured from the EMRs. The morbidity data were based on hospital reports, which ensured its accuracy. Socioeconomic variables were obtained from Statistics Norway, further assuring the validity of the study.

However, the study has some limitations. It is not known why 27.4% of the practices declined to participate. The cross-sectional design limits the potential for exploring explanatory factors for differences in prevalence and intermediary outcomes.

### Comparison with existing literature

The prevalence of CHD among patients with T2DM in the present study is comparable to findings from Sweden^16^ and from Cleveland, US.^17^ However, in a recent US multicentre register study with 575 000 patients with diabetes,^18^ the prevalence of CHD was substantially higher (36.3%), possibly reflecting a more selected population as several centres represented cardiology specialists.

The distribution of CHD and stroke by age, sex, and ethnic group in the present study is in line with other findings.^19,20^

The results regarding lipids are slightly better than found in secondary care in Europe, where 80% of CHD patients with T2DM had LDL >1.8 mmol/l.^21^ In a recent Norwegian post-myocardial infarction study, mean LDL-cholesterol was 2.1 mmol/l and 57% did not reach the target for LDL-cholesterol.^15^

More patients from the present study had SBP <140 mmHg than in the Euroaspire III study (78% versus 28%),^21^ but the findings were comparable to recent results from the large US-based Diabetes Collaborative Registry.^18^

### Implications for research and practice

The finding that CHD often preceded T2DM by several years calls for further research into the relationship between CHD and T2DM, and supports screening for diabetes among patients with CHD, such as measuring HbA1c at the first event of CHD or stroke, and when CHD risk is assessed.^22^

Reasons for not taking statins among people with high risk for CVD events needs more qualitative investigation, and have implications for guidelines. Stricter treatment targets for intermediate outcomes need to be justified by a proper balance between benefits and harms, which represents a challenge in clinical practice. The target for LDL-cholesterol is to some extent arbitrary and GPs may feel that the target is too ambitious for some patients.

The proportion of patients reaching all targets will inevitably decline for every new target that is introduced, and likewise when targets for intermediate outcomes are lowered, if not counteracted by better support for self-management and compliance, including drugs with fewer side effects.

The increased prevalence of CHD and stroke, and younger age at diagnosis, among South Asians together with the fact that glycaemic control in South Asians is inferior compared with that of ethnic Norwegians, highlights the need for special attention towards this group.

Clinically, the groups with the highest blood pressure (SBP >150 mmHg) have a clustering of risk factors and are in need of more effective preventive efforts. Lifestyle interventions — including strategies to support smoking cessation, intensified drug treatment, and support for better compliance — might be needed for a substantial proportion of these high-risk patients.^23^

The diagnosis of CHD preceded the diagnosis of T2DM in half of the patients. Immigrants from South Asia deserve special attention as they have inferior glycaemic control and increased prevalence of CHD at a younger age than the majority population. There is a potential for improvements in secondary prevention, through more prescriptions of lipid-lowering drugs, smoking cessation, and better care for people with multiple elevated risk factors, including high blood pressure.
